# Microbiota and Mitochondrial Sex-Dependent Imbalance in Fibromyalgia: A Pilot Descriptive Study

**DOI:** 10.3390/neurolint15030055

**Published:** 2023-07-12

**Authors:** Jorge A. Ramírez-Tejero, Elena Durán-González, Antonio Martínez-Lara, Laura Lucena del Amo, Isabel Sepúlveda, Andrés Huancas-Díaz, Marco Carvajal, David Cotán

**Affiliations:** 1 Pronacera Therapeutics S.L., 41015 Sevilla, Spain; jorgearamirez@sinae.es (J.A.R.-T.); e.duran@pronacera.com (E.D.-G.); a.martinez@pronacera.com (A.M.-L.); llucdel@alu.upo.es (L.L.d.A.);; 2 Instituto de Medicina Funcional e Integral de Perú, Lima 15073, Peru

**Keywords:** fibromyalgia, microbiota, mitochondria, intestinal dysbiosis

## Abstract

Fibromyalgia is a widespread chronic condition characterized by pain and fatigue. Among the long list of physiological disturbances linked to this syndrome, mitochondrial imbalance and oxidative stress stand out. Recently, the crosstalk between mitochondria and intestinal microbiota has caught the attention of biomedical researchers, who have found connections between this axis and several inflammatory and pain-related conditions. Hence, this pilot descriptive study focused on characterizing the mitochondrial mass/mitophagy ratio and total antioxidant capacity in PBMCs, as well as some microbiota components in feces, from a Peruvian cohort of 19 females and 7 males with FM. Through Western blotting, electrochemical oxidation, ELISA, and real-time qPCR, we determined VDAC1 and MALPLC3B protein levels; total antioxidant capacity; secretory immunoglobulin A (sIgA) levels; and *Firmicutes/Bacteroidetes*, *Bacteroides/Prevotella,* and *Roseburia/Eubacterium* ratios; as well as *Ruminococcus* spp., *Pseudomonas* spp., and *Akkermansia muciniphila* levels, respectively. We found statistically significant differences in *Ruminococcus* spp. and *Pseudomonas* spp. levels between females and males, as well as a marked polarization in mitochondrial mass in both groups. Taken together, our results point to a mitochondrial imbalance in FM patients, as well as a sex-dependent difference in intestinal microbiota composition.

## 1. Introduction

Fibromyalgia (FM) is a widespread chronic condition first introduced in the WHO- ICD in 1992 and currently coded in WHO ICD-11 as MG-30.01. Its course demonstrates two main symptoms: widespread chronic pain and long-lasting fatigue. Nonetheless, a plethora of physical and psychological traits have been linked to this chronic disease, with those affecting the musculoskeletal and nervous systems standing out [1]. Additional common features of FM, such as joint stiffness, muscle twitches, restless sleep, depression, stress, and anxiety, strongly affect the quality of life of these patients, who represent 6% of the global population, most of them middle-aged women from urban areas [2]. Despite the high prevalence, no effective molecular diagnosis for FM has been developed to date. Clinical self-questionnaires, such as the Fibromyalgia Impact Questionnaire (FIQ) and Symptoms Scale (SS), are combined with a physical evaluation of pain through the Widespread Pain Index (WPI), the established positive diagnostic threshold being WPI ≥ 7 and SS ≥ 5 or WPI between 3 and 6 and SS ≥ 9 [3]. This vague diagnostic is supported by a confusing etiopathogenesis that has been deeply reviewed in the last two decades and recently summarized by Sarzi-Puttini et al. [4]. These authors grouped all the related symptoms into two differentiated groups: cardinal and common features. While the first category corresponds to those symptoms that are always present in patients, such as generalized pain, fatigue, and sleep disturbances, the second includes those symptoms shared with other comorbidities, such as psychological affections, regional pain, and hyperalgesia/allodynia. Current screening tools are based on clinical questionnaires that only determine physical and psychological performance, as well as the presence of widespread pain [4], due to the lack of biomarkers. For this reason, the research on biomarkers for FM has focused on an endless list of putative candidates related to clinical imaging techniques, genetic alterations, the immune system, mitochondrial metabolism, hormones and neurological signaling [5]. Although it is difficult to clarify such a controversial topic, it makes sense to deepen research on the gut–mitochondria axis, since a profound lack of energy in the muscle and central nervous system is reported by FM patients [6,7], and this axis has a crucial role in nutrient digestion and energy production.

Microbiota are ubiquitous in the human body. Several genera from the Bacteria, Archaea, and Eukarya kingdoms colonize not only the outer parts of the body but also the inner ones. In this sense, intestinal microbiota are one of the most abundant and diverse communities in the body [8], with a direct impact on the maintenance of health and energy balance. Although this community is quite unstable in early life, intestinal microbiota are more balanced and well-documented in late adulthood, a period of life when only long-term factors, such as weight gain, travel, or diseases, can disrupt their stability [9]. This equilibrium depends on the distribution of the most abundant phyla in the intestinal microbiota: *Firmicutes*, *Bacteroidetes*, *Actinobacteria*, *Verrucomicrobia,* and *Proteobacteria*. The measurement of these phyla/genera and their ratios are useful to determine intestinal microbiota health (e.g., *Firmicutes*/*Bacteroidetes* and *Bacteroides/Prevotella*) [10]. When the distribution of these phyla/genera is altered, intestinal dysbiosis appears and triggers a negative feedback loop that underlies a broad spectrum of diseases located not only in the intestinal system but also in the immune [11], cardiovascular [12,13], and musculoskeletal [14,15] systems, paving the way for the appearance of allergies, cardiovascular pathologies, and a broad spectrum of rheumatic diseases, respectively. Strikingly, the vast majority of these effects are closely related to FM symptoms, as reported in the EPIFFAC study [16]. Thus, some authors have pointed to intestinal microbiota as a main actor in the FM condition [17,18]; they have been proposed as a biomarker [19] and are even emerging as a promising therapeutic target for supplementation and diet [20].

The mitochondria is the powerhouse of the cell. While intestinal microbiota are the first line in food digestion and nutrient processing, the final aim of the entire process is to provide energy to the cells through ATP production—a molecular pathway that takes place in the mitochondria. Thus, even though this organelle is heterogeneously present in human cells, it is especially abundant in those tissues with higher energy demand, such as the muscles and brain [21], both strongly affected by the etiology of FM [22]. In fact, several authors have highlighted the implication of mitochondrial imbalance in patients affecting both mtDNA and several mitochondrial pathways, such as the electron transport chain, tricarboxylic acid cycle, and fatty acid beta-oxidation [23,24,25,26,27,28]. As a result, FM patients have shown an increased level of oxidative stress [29,30] that, later, causes difficulties at multiple levels, such as mitochondrial biogenesis and selective mitochondrial autophagy (mitophagy). Furthermore, the crosstalk between mitochondria and microbiota, also defined as the gut–mitochondria axis, is involved in inflammation [31], a hallmark of FM.

Hence, the main objective of this retrospective, observational pilot study was to characterize the composition of the intestinal microbiota, as well as the mitochondrial balance, to explore the differences between male and female FM patients.

## 2. Materials and Methods

### 2.1. Patients

This retrospective, observational pilot study was carried out on 26 patients from the Institute for Functional Medicine (IFM) in Lima (Peru). The medical records were coded and anonymized to comply with Regulation 2016/079 of the European General Data Protection Regulations. The patients who were considered for this study had already been diagnosed with primary FM by an experienced practitioner according to the 2010 criteria of the American College of Rheumatology [3]. Inclusion criteria were based on age (only adult patients were included) and lifestyle habits (smokers and morbidly obese patients were not allowed to participate because of the deleterious effect of both). All the patients signed an informed consent form to participate in this study, which was approved by an ethics committee and conducted according to the Declaration of Helsinki from the World Medical Association.

### 2.2. Health Questionnaires

The patients filled out the clinical symptoms sheet, a self-questionnaire from the Cleveland Clinic Center for Functional Medicine and IFM (Cleveland, OH, USA) which is used to assess the impact of the condition on overall health (Supplementary File S1). Statistical analysis was performed using the statistical software STATA v.15^®^ (StataCorp 2015, Stata Statistical Software, College Station, TX, USA). The IFM questionnaire has 15 items describing physical and psychological health. The answers refer to the feelings that the patient has experienced during the last two weeks, and the rating scale ranges from 0 to 4 points: 0 means “never or almost never experienced the symptom”; 1 means “occasionally experienced it, but the effect is not serious”; 2 means “experienced it occasionally and the effect is severe”; 3 means “experienced it frequently, but the effect is not serious”; and 4 means “frequently suffered it and the effect is serious”. The final score is divided into five groups: from 0–10 as “good health”; from 10–20 as “mild dysfunction”; from 20–50 as “moderate dysfunction”; from 50–100 as “severe dysfunction”, and more than 100 as “critical dysfunction”.

### 2.3. Determination of Mitochondrial Mass and Total Antioxidant Capacity

**Figure 1:**
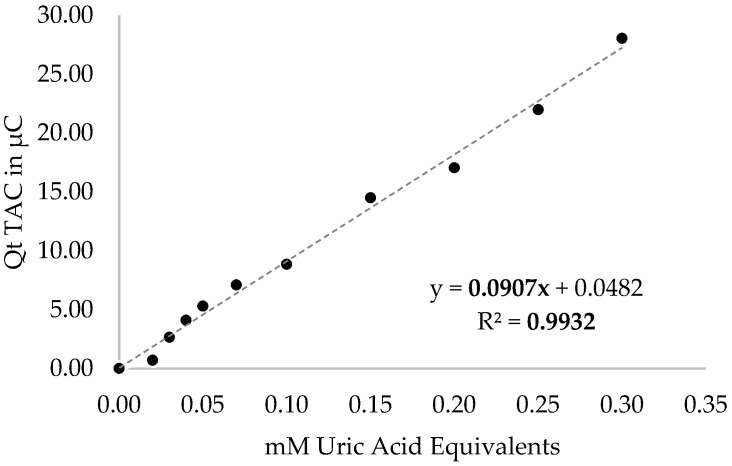
Pattern of uric acid used in eBQC TAC measurement.

Peripheral blood samples were collected from patients in the early morning using a BD Vacutainer^®^ Safety-Lok™ blood collection set and two BD Vacutainer^®^ Lithium Heparin Tubes (Becton Dickinson, Franklin Lakes, NJ, USA). One of the tubes was used to determine the mitochondrial ratio and total antioxidant capacity (TAC), while the other one was used for peripheral blood mononuclear cells protein extraction (PBMC). For the isolation of PBMCs, 5 mL of whole blood was mixed with 45 mL of ammonium chloride lysis buffer 0.17 M (Thermo Fisher Scientific, Waltham, ME, USA). After shaking for 5 min at room temperature, the tubes were centrifuged for 10 min at 600 FCR, recovering the isolated PBMC pellet. Cells were then lysed with a buffer containing 0.32 M sucrose, 10 mM tris-HCl, 5 mM MgCl2, Triton X-100 1%, and proteinase inhibitor. After a 20 min incubation period on ice, samples were sonicated for better cell lysis. The subsequent whole protein quantification was performed using the Lowry method at 750 nm. The mitochondrial mass was determined using the Western blotting technique in PBMC, as described by Martínez-Lara et al. [28] Hence, mitochondrial mass was evaluated through immunodetection of voltage-dependent anion-selective channel protein 1 (VDAC1; UniProt Accession P21796), while mitophagy was measured through microtubule-associated proteins 1A/1B light chain 3B (MAP1LC3B; UniProt Accession Q9GZQ8) using specific monoclonal antibodies (Abcam, Cambridge, UK). For TAC measurement, an e-BQC lab device (Bioquochem SL, Oviedo, Asturias, Spain) was used. e-BQC lab is an electroanalytical system designed for measuring the antioxidant capacity of biomedical samples [32,33]. The device provided TAC values in terms of electric charge units (Q, µC). The e-BQC lab TAC results were transformed into uric acid equivalents (mM) using a standard curve with a wide range of uric acid dilutions (0/0.02/0.03/0.04/0.05/0.07/0.10/0.15/0.20/0.25/0.30 mM), as shown in Figure 1.

### 2.4. Microbiota Analysis

Fecal samples were collected with a DANASTOOL Sample Collection MICROBIOME Kit (DANAGENE, Barcelona, Spain) containing a DNA stabilization solution for sample shipment to avoid degradation. DNA extraction was performed in 0.5–1 g of the sample with a DANAGENE Microbiome Fecal DNA kit (DANAGENE, Barcelona, Spain) via a cetyltrimethylammonium bromide (CTAB) buffer-based procedure. Briefly, a portion of the sample was placed into a 2 mL Eppendorf^®^ microtube with 1 mL of CTAB. Then, the mixture was roughly homogenized by pipetting to avoid foam and incubated for 10 min at 70 °C. Next, homogenization of the sample took place on a Vortex Genie 2 for 10 min at maximum speed, and, after that, it was centrifuged at 14,000 rpm for 5 min. Then, 600 µL of the supernatant was transferred to a 1.5 mL Eppendorf^®^ microtube with 200 µL of EC buffer to remove contaminants, and subsequently mixed in vortex. The following steps included 5 min of incubation at 4 °C and centrifugation at 14,000 rpm for 5 min in order to transfer 500 µL of supernatant into a new 1.5 Eppendorf^®^ microtube. Before transferring the liquid to the MicroSpin column, Proteinase K incubation was performed (25 µL, 70 °C, 10 min). The obtained product was placed into a reservoir of the MicroSpin column for the binding, washing, and elution steps, with rounds of centrifugation of 10,000 rpm for 60 s. The quality and purity of the extracted DNA were assessed by absorbance at 260 nm with a µDro Duo plate in a Multiskan SkyHigh Plate Reader (Thermo Fisher Scientifics, Waltham, ME, USA). Bacterial abundances were quantified by real-time quantitative PCR and then used to calculate the ratios of *Firmicutes/Bacteroidetes*, *Bacteroides/Prevotella*, and *Roseburia/Eubacterium*, as well as the presence of *Ruminococcus*, *Pseudomonas*, and *Akkermansia muciniphila*. The primer designs are described in Table 1.

Additionally, intestinal secretory IgA was determined with an ELISA kit (Eagle Bioscience, Amherst, NH, USA), following the recommendations of the manufacturer.

## 3. Results

### 3.1. Demographic Profile of Patients

Of the patients included in the study, 19 (73%) were female and 7 (27%) were male. The age range was between 43 and 55, with a mean age of 47 years. Patients did not have any nutritional habits (vegetarian, vegan, etc.), nor any therapeutic regimens (ketogenic diets, elimination diets, or similar). More than half of the patients (62%) engaged in aerobic exercise (walking, running, swimming, or stationary cycling) or mild vigorous exercise (yoga, Pilates) for at least 1 h 3 times per week. Of these patients, 62% did not undertake any psychological or emotion-focused therapy. Additionally, up to 68% of the patients were consuming non-steroidal anti-inflammatory drugs (NSAIDs). As Table 2 demonstrates, 82% of the patients presented with slight to critical dysfunction. The categorical variables, such as sex, type of nutrition, physical exercise, and drug consumption, are expressed by count. For numerical variables such as age, the mean and standard deviation are expressed in brackets, and the *p*-values, as calculated using Student’s *t*-test, are shown.

### 3.2. Mitochondrial Mass and Total Antioxidant Capacity

**Figure 2:**
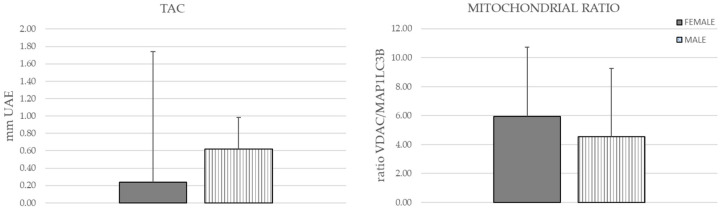
Total antioxidant capacity and mitochondrial ratio comparison between male and female patients.

The mitochondrial ratio was imbalanced compared to the mitochondrial mass, showing higher VDAC protein levels in both groups (Figure 2). Similarly, the TAC mean showed no statistically significant differences between male and female patients (Figure 2).

### 3.3. Microbiota Composition

**Figure 3:**
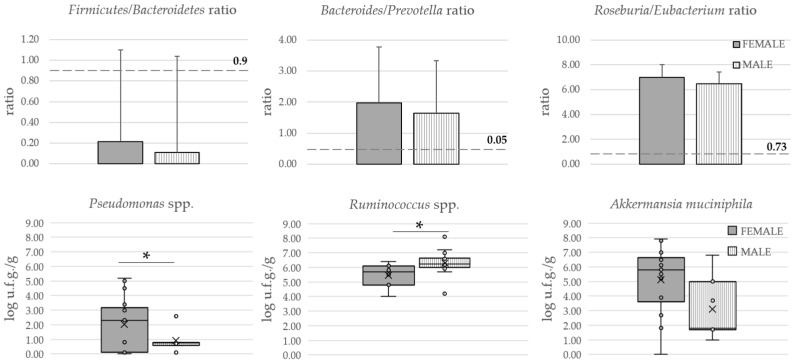
Intestinal microbiome measurements. * *p*-value < 0.05. For the *Firmicutes*/*Bacteroidetes*, *Bacteroides*/*Prevotella*, and *Roseburia*/*Eubacterium* plots, dashed lines represent the minimal ratio found in healthy volunteers across the bibliography [40,41,42]. For the *Pseudomonas*, *Ruminococcus*, and *Akkermansia* graphs, box-and-whisker plots show the median (horizontal line inside the box), the mean (crosses inside the box), the lower and upper quartiles (lower and upper box limits), and the maximum and minimum values (the whiskers).

None of the ratios showed differences between male and female patients. However, female patients showed higher levels of *Ruminococcus* spp. and *Pseudomonas* spp. (Figure 3).

**Figure 4:**
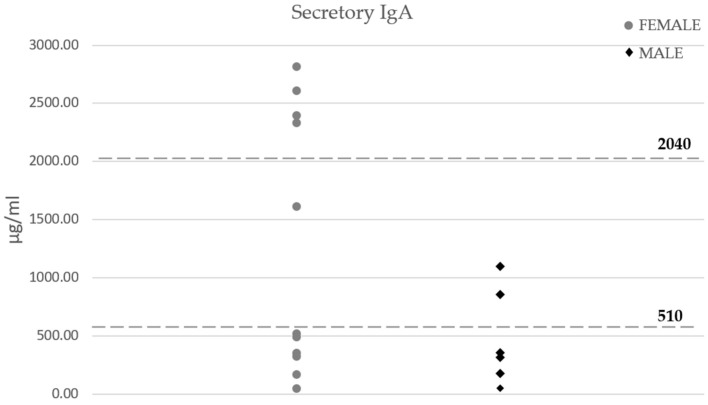
Secretory IgA levels determined with ELISA. The dashed line represents the normal range according to the manufacturer.

The mean secretory IgA levels in women were double compared with those found in men, although there were no statistically significant differences. Interestingly, most of the patients (85%) showed out-of-range levels in both groups (Figure 4).

## 4. Discussion

The current observational pilot study analyzed mitochondrial and microbiota molecular markers in a Peruvian cohort of female and male FM patients. Although there were no differences in the mitochondrial mass or the TAC, the results showed *Pseudomonas* and *Ruminococcus* to be differentially expressed bacteria between both groups.

Chronic conditions are usually difficult to tackle, not only because of the numerous factors that contribute to their development but also due to the heterogeneous group of patients. FM is one of these diseases. In fact, recent publications have proposed different groups of FM patients according to their pain symptoms [43]. Despite this, several research groups have been trying to find molecular biomarkers that could be useful for the diagnostics, treatment tracking, and classification of these patients. In this sense, FM has been traditionally linked to oxidative stress and mitochondria. Several authors have found mitochondrial dysfunction, mtDNA mutations, and different oxidative stress markers in FM patients [23,24,25,26,27,28,30,44]. Later, these problems underlie to chronic and neuropathic pain, as well as inflammation, not only in FM but also in a plethora of complex syndromes [45,46,47,48,49]. In line with this hypothesis, our results showed VDAC1 protein levels that were higher than MAP1LC3B levels in female and male patients. VDAC1 is a mitochondrial membrane porin that is the predominantly expressed form in this organelle [50], while MAP1LC3B is closely related to mitophagy since it is the first protein to trigger the molecular signaling that ends in mitochondrial autophagy [51,52]. These results suggest that FM patients could have a sex-independent marked polarization to mitochondrial accumulation and a subsequent unbalanced oxidative metabolism, even though the lack of healthy volunteers in our study makes it difficult to support the hypothesis that points to mitochondria and oxidative stress as hallmarks of this syndrome. Additionally, the high number of patients consuming drugs such as NSAIDs and analgesics in our cohort might be a confounding factor in determining both parameters, since a huge number of pharmacological therapies affect mitochondrial function [53]. Indeed, these drugs not only influence the mitochondria and oxidative metabolism enzymes (such as COX-1 and COX-2), but also on gastrointestinal homeostasis, especially in microbiota [53]. Hence, as discussed later in this section, the higher number of female patients taking NSAIDs might have an influence on both areas. To solve this, further research with more patients and better-defined drug consumption is needed.

Recently, a growing number of researchers have been attempting to find connections between microbiota and many chronic illnesses. In fact, the interplay between microbiota and mitochondria is rising as a promising diagnostic and therapeutic target for pain, neurodegeneration, and metabolic affections such as type 2 diabetes and obesity, among others [54,55,56]. In particular, chronic pain-related conditions are closely connected to gastrointestinal disturbances and the gut–brain axis [57]. Concretely, several neurotransmitters and metabolites related to pain processing and inflammation are produced in the intestine by a long list of microbes that colonize it [58,59,60]. Therefore, this could be a connection to be analyzed in chronic pain-related conditions such as FM. Our results did not show statistically significant differences between female and male patients in the three microbiota ratios which we analyzed, but the *Firmicutes*/*Bacteroidetes* ratio was below the normal range found in healthy volunteers in a previous study [40]. This ratio seems to positively correlate with BMI and type 2 diabetes [61,62,63], although different cohorts of individuals from different countries showed opposite results [40]. Nonetheless, both genera exert interesting metabolic activities which could be key to their health impact. *Firmicutes* and *Bacteroidetes* have huge gene clusters coding for P450 monooxygenases, ferredoxins, and other secondary metabolites with relevant roles in host metabolism, mostly in oxidative pathways. These clusters are more abundant in *Bacteroidetes* species, and have been proposed as factors responsible for health changes attributed to this ratio [64]. Given that several FM studies have shown oxidative stress and mitochondrial dysfunction markers in these patients [23,24,25,26,27,28,30,49], the low *Firmicutes*/*Bacteroidetes* ratio found in our cohort could somehow be linked to mitochondrial imbalance, which was also present in this group. Nonetheless, as previously pointed out, our patients are widely exposed to pharmacological treatment, with a relevant number of participants using NSAIDs or analgesic treatment, especially women. A plethora of authors have highlighted the strong impact of NSAID treatments on intestinal homeostasis and microbiota, as widely reviewed by Maseda and Ricciotti as well as Zádori et al., among others [65,66]. For instance, the use of well-known NSAIDs such as indomethacin [67,68,69,70], diclofenac [71], or ibuprofen [72] has been shown to influence the abundance of *Bacteroides* in different cohorts of human subjects and animal models. Surprisingly, these are some of the most used NSAIDs among FM patients, so microbiota alterations within this pathology could be linked to the treatment. A detailed discussion regarding NSAIDs' full repertoire of microbiota-related effects can be found in the review by Zadori et al. [66]. As a result, these findings provide a starting point to shed some light on the differences in microbiota composition between FM patients and healthy volunteers.

Focusing on the specific genera, while *Akkermansia muciniphila* showed no differences, *Ruminococcus* spp. and *Pseudomonas* spp. did demonstrate statistically significant differences between female and male patients with FM. *Ruminococcus* spp., an anaerobic gram-positive coccus, was lower in women. This genus was found to be positively correlated with BMI [73], and, in our cohort, men had a slightly higher BMI. Thus, BMI itself could be responsible for this difference between the two groups. *R. gnavus* has been identified as an inflammatory LPS-producing bacterium that is able to increase TNF-alpha production [74]. Since low-grade inflammation is a common feature of FM, the role of *Ruminococcus* spp. in this condition should be studied. In contrast, Yunus et al. [75] demonstrated that male FM patients had lower tender points, pain and gastrointestinal symptoms than women. According to this, the differences found in these symptoms could not be supported by *Ruminococcus* spp. differences, although further research is needed. Strikingly, this genus was found to be decreased in feces from an animal model of endometriosis [76], a pain-related condition that has been identified as a comorbidity of FM [77]. In line with this, the *Ruminococcaceae* family was found to be negatively correlated with enterocyte apoptosis and IL-6 levels in mice, indicating a potential protective role of this bacterium against intestinal inflammation [78]. Furthermore, *Ruminococcus* has been studied as a key bacterium in estrobolome, the group of enzymes and metabolic reactions that depend on microbiota and that impact estrogens levels. In fact, β-glucuronidase from *Ruminococcus* has shown its capability to reactivate estrogens in the gut lumen, allowing them to re-enter the bloodstream in their active form [79]. Similarly, the G-protein-coupled estrogen receptor has been proposed as a differential biomarker between FM patients and healthy controls, with higher levels in patients being negatively correlated with prolactin levels [80]. Taken together, these results highlight the promising role of hormonal imbalances and the abundance of *Ruminococcus* in the differential prevalence of FM between males and females, since lower levels of this bacterium might have an indirect impact on inflammation and the well-known differences in estrogen-related pain signaling between males and females.

*Pseudomonas* spp. also showed statistically significant differences in our result, proving to be lower in male patients. *Pseudomonas* are an aerobic group of gram-negative bacilli that act mainly as opportunistic microorganisms, causing pathogenic disturbances in the intestine [81]. Taking into account that gastrointestinal symptoms seem to be more frequent in females [75], *Pseudomonas* could be one of the genera responsible for this situation. In fact, it has been demonstrated that, in chronic fatigue syndrome, serum IgA anti-*Pseudomonas* LPS increases compared to healthy controls, and their levels positively correlate with the clinical symptoms of these patients [82]. Secretory IgA has been traditionally studied at a gastrointestinal level as a master regulator of intestinal microbiota, being responsible for the immune responses to its changes [83]. This antibody did not show differences between female and male feces in our FM cohort, but 85% of them had out-of-range sIgA concentrations. Although congenital immunodeficiency and other influential factors must be discarded, these changes could be behind the shifts in microbiota between the two groups of Peruvian patients. Maes et al. [82] suggested that antioxidants may be useful in chronic fatigue syndrome to repair the intestinal barrier, whose increased permeability facilitates the blood circulation of LPS. Whether this intervention could help FM patients or not to control intestinal dysbiosis and the intestinal barrier needs to be addressed. Moreover, further research is needed in order to understand the influence of sex, drugs, nutrition, supplements, and life habits on gastrointestinal health in the FM context.

## 5. Conclusions

Taken together, our results suggest that the microbiota–mitochondria axis can help in the diagnostics, treatment tracking, and classification of FM patients. sIgA, *Ruminoccocus* spp., and *Pseudomonas* spp. might be useful markers not only to diagnose FM, but also to differentiate between male and female patients. However, to properly define the roles of these markers and the microbiota–mitochondria axis, further research with a higher and better-defined number of patients is necessary.

## Figures and Tables

**Table 1:** Primer designs for the characterization of intestinal microbiota.

Bacteria	Primer Pair	References
*Akkermansia muciniphila*	AkkF, AkkR	[10,34,35,36,37,38,39]
*Bacteroides* spp.	BacF ^1^, BacR ^1^	
*Bacteroidetes* spp.	BatsF, BatsR	
*Eubacterium* spp.	EuF ^A^, EuR ^A^	
*Firmicutes* spp.	FirmF, FirmR	
*Prevotella* spp.	PrevF ^2^, PrevR ^2^	
*Pseudomonas* spp.	PseF ^A^, PseR ^A^	
*Roseburia* spp.	RosF ^A^, RosR ^A^	
*Ruminococcus* spp.	RumF ^3^, RumR ^3^	
All bacteria	AllF ^A^, AllR ^A^	

^1^*Bacteroides* spp. targeted *B. acidifaciens*, *B. caccae*, *B. clarus*, *B. faecalis*, *B. intestinalis*, *B. oleiciplenus*, *B. rodentium*, *B. stercoris*, *B. uniformis*. ^2^ *Prevotella* spp. targeted *P. corporis*, *P. buccae*, *P. buccalis*, *P. dentalis*, *P. dentasini*, *P. denticola*, *P. enoeca*, *P. histicola*, *P. intermedia*, *P. loescheii*, *P. maculosa*, *P. oralis*, *P. phocaeensis*. ^3^ *Ruminococcus* spp. targeted mainly, *R. gnavus*. ^A^ Species targeted by these primers can be checked in the list of references.

**Table 2:** Demographic and health habit profiles of patients.

Variable	Outcome	Male (*n* = 7)	Female (*n* = 18)	*p*-Value
Age		45.43 (±8.96)	50.28 (±10.21)	0.26
Body Mass Index (BMI)		26.26 (±3.31)	24.97 (3.07)	0.39
IFM questionnaire score		51.80 (±15.87)	62.17 (±35.37)	0.06
Nutrition	Not controlled	7	18	n.a. *
	Under control	0	0	
Physical exercise	None	3	7	n.a. *
	Yes	4	11	
Psychological therapy	None	4	7	n.a. *
	Yes	3	11	
Drugs ^1^	NSAIDs	4	14	n.a. *
	Antidepressants	4	10	
	Neuropathic drugs	1	7	
	Analgesics	5	15	
	Antibiotics ^2^	2	2	
	Probiotics ^2^	2	5	

^1^ Refers to the moment in which the samples were taken, except for antibiotics and probiotics, which refer to the last 3 months. ^2^ Antibiotic and probiotic treatments were suspended at least 15 days before sampling. * Not applicable.

## Data Availability

Data sharing is not applicable.

## Supplementary Materials

The following supporting information can be downloaded at: https://www.mdpi.com/article/10.3390/neurolint15030055/s1, Supplementary File S1: Cleveland Clinic Center for Functional Medicine and IFM (Cleveland, OH, USA) Health Questionnaire, used to assess the overall health self-perception of patients.
