# Supplementary material for: Microbiota and Mitochondrial Sex-Dependent Imbalance in Fibromyalgia: A Pilot Descriptive Study
Source: Neurol Int. 2023 Jul 12;15(3):868–80. doi: 10.3390/neurolint15030055 (PMC10366818; doi:10.3390/neurolint15030055)
Supplement: Supplementary file 1 [file neurolint-15-00055-s001.zip › neurolint-2419654-supplementary.pdf]

THE INSTITUTE FOR FUNCTIONAL MEDICINE®

Medical Symptoms Questionnaire (MSQ)

Patient Name________________________________ Date______________

Rate each of the following symptoms based upon your typical health profile for the past 14 days.

Point Scale

0 – ***Never or almost never*** have the symptom
1 – ***Occasionally*** have it, effect is ***not severe***
2 – ***Occasionally*** have it, effect is ***severe***
3 – ***Frequently*** have it, effect is ***not severe***
4 – ***Frequently*** have it, effect is ***severe***

HEAD

_______ Headaches
_______ Faintness
_______ Dizziness
_______ Insomnia

Total _______

EYES

_______ Watery or itchy eyes
_______ Swollen, reddened or sticky eyelids
_______ Bags or dark circles under eyes
_______ Blurred or tunnel vision
(Does not include near or far-sightedness)

Total _______

EARS

_______ Itchy ears
_______ Earaches, ear infections
_______ Drainage from ear
_______ Ringing in ears, hearing loss

Total _______

NOSE

_______ Stuffy nose
_______ Sinus problems
_______ Hay fever
_______ Sneezing attacks
_______ Excessive mucus formation

Total _______

MOUTH/THROAT

_______ Chronic coughing
_______ Gagging, frequent need to clear throat
_______ Sore throat, hoarseness, loss of voice
_______ Swollen or discolored tongue, gums, lips
_______ Canker sores

Total _______

SKIN

_______ Acne
_______ Hives, rashes, dry skin
_______ Hair loss
_______ Flushing, hot flashes
_______ Excessive sweating

Total _______

HEART

_______ Irregular or skipped heartbeat
_______ Rapid or pounding heartbeat
_______ Chest pain

Total _______

MEDICAL SYMPTOMS QUESTIONNAIRE (MSQ)

LUNGS

______ Chest congestion
______ Asthma, bronchitis
______ Shortness of breath
______ Difficulty breathing

Total ______

DIGESTIVE TRACT

______ Nausea, vomiting
______ Diarrhea
______ Constipation
______ Bloated feeling
______ Belching, passing gas
______ Heartburn
______ Intestinal/stomach pain

Total ______

JOINTS/MUSCLE

______ Pain or aches in joints
______ Arthritis
______ Stiffness or limitation of movement
______ Pain or aches in muscles
______ Feeling of weakness or tiredness

Total ______

WEIGHT

______ Binge eating/drinking
______ Craving certain foods
______ Excessive weight
______ Compulsive eating
______ Water retention
______ Underweight

Total ______

ENERGY/ACTIVITY

______ Fatigue, sluggishness
______ Apathy, lethargy
______ Hyperactivity
______ Restlessness

Total ______

MIND

______ Poor memory
______ Confusion, poor comprehension
______ Poor concentration
______ Poor physical coordination
______ Difficulty in making decisions
______ Stuttering or stammering
______ Slurred speech
______ Learning disabilities

Total ______

EMOTIONS

______ Mood swings
______ Anxiety, fear, nervousness
______ Anger, irritability, aggressiveness
______ Depression

Total ______

OTHER

______ Frequent illness
______ Frequent or urgent urination
______ Genital itch or discharge

Total ______

Grand Total ______
